# Transcriptomic Convergence in Autism Spectrum Disorder: Synaptic, Immune‐Glial and RNA‐Regulatory Axes in the Human Cerebral Cortex

**DOI:** 10.1002/jdn.70157

**Published:** 2026-07-09

**Authors:** Ruslan Kurmashev

**Affiliations:** ^1^ Munster Technological University Cork Ireland

**Keywords:** alternative splicing, autism spectrum disorder, cortical transcriptomics, immune‐glial activation, neuronal and synaptic dysregulation, post‐mortem cerebral cortex, RNA processing, single‐cell transcriptomics

## Abstract

Autism spectrum disorder (ASD) arises from highly heterogeneous genetic and developmental liabilities, raising the question of whether this heterogeneity converges on shared molecular programmes in the human cerebral cortex. This structured review, based on systematic database searching and narrative synthesis, examined that question specifically in human post‐mortem cortical transcriptomic studies. PubMed, Scopus and Europe PMC were searched from 1 January 2009 to 6 May 2026, and 43 studies met the final eligibility criteria. Across the available literature, the evidence does not support a single invariant cortical transcriptomic signature in ASD. Rather, the most consistent signal indicates non‐uniform convergence on reduced neuronal and synaptic expression together with increased immune‐glial programmes. A substantial additional body of evidence implicates dysregulation of transcript‐regulatory processes, particularly in studies interrogating alternative splicing and related RNA‐processing mechanisms. Cell‐resolved datasets further suggest that these abnormalities are concentrated within defined neuronal and glial populations rather than being distributed uniformly across the cortex. By contrast, mitochondrial and broader metabolic alterations are supported less consistently and are better interpreted as conditional or secondary features of cortical pathology than as equally well‐established core axes. Interpretation of these findings is constrained by the structure of the evidence base itself. Only 9 of the 43 included studies were judged to provide direct support for the central convergence question, and only 13 were based on primary independent cohorts; much of the literature relies on dataset reuse, regionally restricted sampling and heterogeneous analytical platforms. Collectively, human cortical transcriptomic studies in ASD support a model of partial, context‐dependent convergence on a limited set of biological programmes, rather than a single stable molecular lesion.

## Graphical abstract

Across 43 human post‐mortem cortical transcriptomic studies, ASD shows recurrent but non‐uniform convergence on reduced synaptic‐neuronal signalling, increased immune‐glial activity and RNA‐regulatory dysregulation. Metabolic and mitochondrial changes are less consistently supported and appear more conditional within the current cortical evidence base.

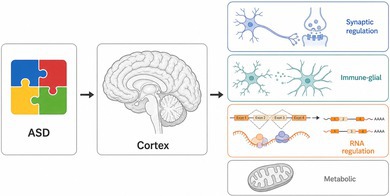

## 1 Introduction

Autism spectrum disorder (ASD) is a heterogeneous neurodevelopmental condition defined behaviourally, but marked by substantial clinical, developmental and biological variability across affected individuals (Lord et al. 2020). Its genetic architecture encompasses both rare high‐impact variants and a substantial contribution from diffuse common polygenic risk, making it unlikely that ASD can be reduced to a single pathogenic mechanism or a single invariant molecular lesion (Grove et al. 2019; Satterstrom et al. 2020). Nevertheless, heterogeneous upstream liabilities may still converge on a restricted set of downstream programmes within cortical systems, particularly those involving neuronal differentiation, synaptic communication and gene regulation (Willsey et al. 2013; Sullivan et al. 2019; Garcia‐Forn et al. 2020; Courchesne et al. 2020).

Transcriptomic analysis provides a useful framework for addressing this problem because it captures downstream molecular programmes after genetic, developmental and cellular influences have been integrated at the tissue level. In ASD, the most informative human evidence has emerged from post‐mortem brain studies, particularly those focused on the cerebral cortex, where foundational bulk analyses first identified coordinated neuronal and synaptic downregulation together with immune‐glial upregulation and altered regional cortical patterning (Voineagu et al. 2011; Gupta et al. 2014; Parikshak et al. 2016; Gandal et al. 2022). More recent single‐cell, single‐nucleus and transcript‐aware studies have refined this framework by localising dysregulation to defined neuronal and glial populations and by showing that ASD‐associated abnormalities extend beyond total gene abundance to alternative splicing, microexon regulation, RNA editing and other post‐transcriptional layers (Irimia et al. 2014; Tran et al. 2019; Velmeshev et al. 2019; Zhang et al. 2023; Wamsley et al. 2024). These advances have improved biological resolution, but they have not resolved the central interpretive challenge: whether ASD exhibits genuine cortical transcriptomic convergence or instead a patchwork of region‐specific, cohort‐specific and method‐specific findings.

Recent reviews have summarised broader ASD transcriptomic and biomarker‐related findings, including narrative overviews of brain transcriptomics and integrative immune‐metabolic syntheses extending beyond cortex‐centred post‐mortem evidence (Meng et al. 2025; Trivedi et al. 2025). Although these broader accounts are useful, they do not fully resolve a narrower question of direct relevance here: which transcriptomic alterations recur specifically in human post‐mortem cortical tissue, and how robust are those signals once study independence, dataset reuse, mixed cortical versus non‐cortical design and methodological heterogeneity are made explicit? This issue matters because apparent consistency in the literature can be inflated when primary studies, public‐dataset reanalyses and summary‐level integrative syntheses are discussed as though they carried comparable inferential weight (Lombardo et al. 2017; Ramaswami et al. 2020; Velmeshev et al. 2020).

This review therefore adopts a cortex‐centred, evidence‐weighted perspective on ASD transcriptomics. The aim is not to argue for a single invariant molecular signature of ASD, but to determine whether recurrent, albeit non‐uniform, biological programmes can be identified across human post‐mortem cortical studies despite substantial regional, developmental, subtype‐related and methodological heterogeneity. Primary weight is assigned to peer‐reviewed human cortical evidence, and the relative support for neuronal/synaptic, immune‐glial and transcript‐regulatory abnormalities is evaluated while making cohort overlap, dataset reuse, platform differences and the interpretive limits of post‐mortem material explicit. Within the final corpus of 43 included studies, only 9 were judged to provide direct support for the core convergence question, and only 13 were based on primary independent cohorts, making critical appraisal essential to any defensible synthesis.

This deliberately conservative scope is intended to sharpen, rather than broaden, the central biological question. Peripheral transcriptomic studies, organoid and stem‐cell systems, animal models and mixed or non‐cortical designs remain useful for context and interpretation, but are not treated here as equivalent to primary human cortical evidence. The question, therefore, is not whether ASD cortex exhibits a single uniform transcriptomic profile, but whether recurrent cortical biological programmes can be identified without overstating consistency across a heterogeneous and only partly independent evidence base.

## 2 Methods

This review was conducted as a structured review with systematic database searching and narrative synthesis. The objective was not to perform a statistical meta‐analysis, but to identify, critically appraise and interpret the strongest available human post‐mortem cortical transcriptomic evidence relevant to ASD while explicitly accounting for study heterogeneity, dataset reuse and methodological limitations.

The formal search window extended from 1 January 2009 to 6 May 2026. Records were identified through systematic searches of PubMed, Scopus and Europe PMC. Search terms combined three concept blocks related to ASD, transcriptomics and brain or cortical tissue, including autism spectrum disorder, autism, ASD, transcriptome, RNA‐seq, gene expression, single‐cell RNA‐seq, single‐nucleus RNA‐seq, alternative splicing, isoform, brain, cortex, cortical, cerebral cortex, post‐mortem and postmortem, with syntax adapted to each database. No additional records contributing to the final evidence set were formally identified through backward citation screening, forward citation screening or manual journal hand‐searching.

**FIGURE 1:**
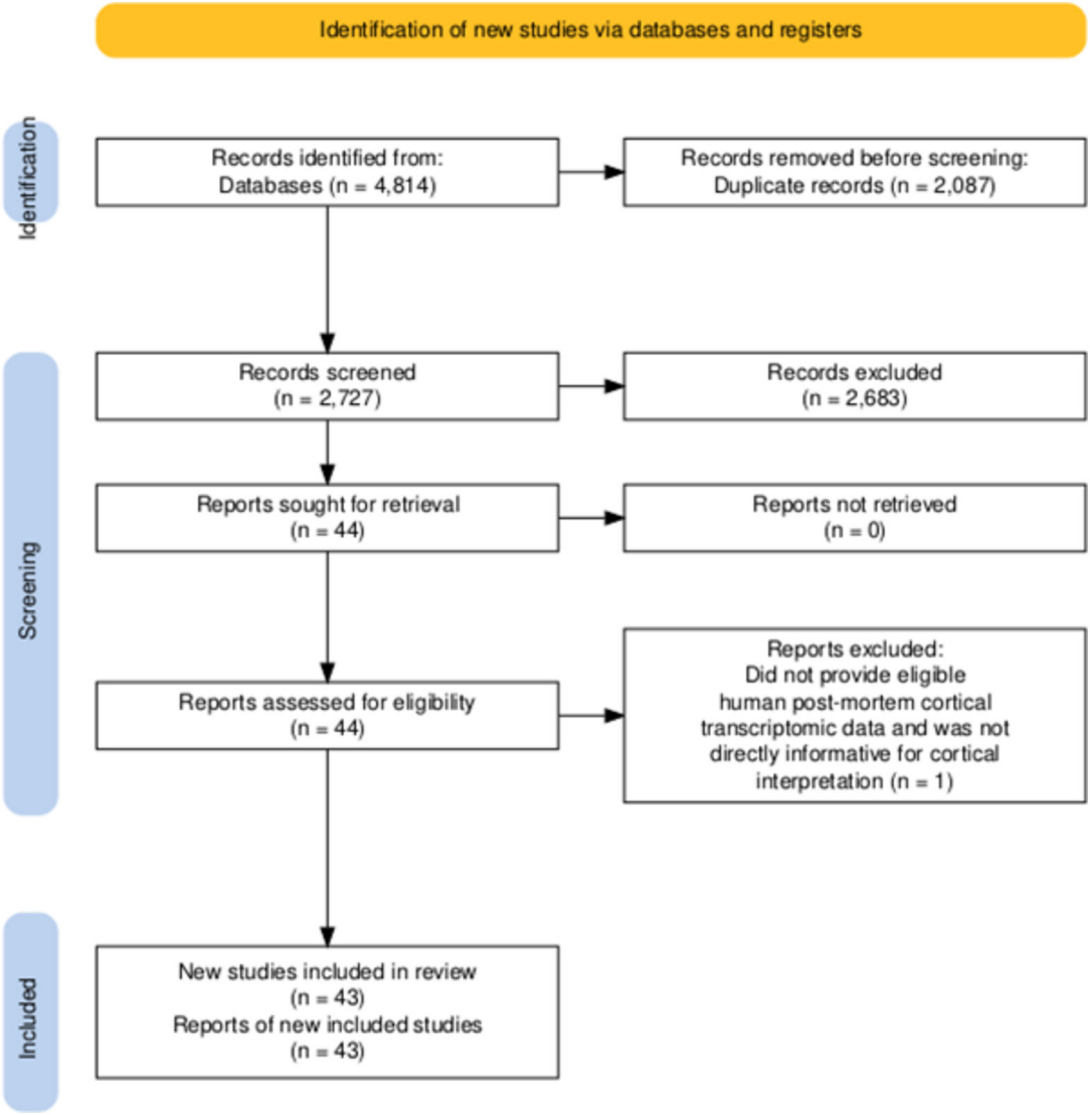
PRISMA 2020 flow diagram of study identification, screening, eligibility assessment and inclusion for the structured review. Records were identified through PubMed (*n* = 1532), Scopus (*n* = 2545) and Europe PMC (*n* = 737). After removal of duplicates (*n* = 2087), 2727 records underwent title and abstract screening, 44 reports were assessed in full text, and 43 studies were included in the final qualitative synthesis (Haddaway et al. 2022).

All records were imported into Rayyan for deduplication, title and abstract screening, full‐text screening and exclusion tracking. Screening was performed by a single reviewer and should therefore be regarded as a methodological limitation of the review process. A total of 4814 records were identified across the three databases, including 1532 from PubMed, 2545 from Scopus and 737 from Europe PMC. After deduplication, 2727 records remained for title and abstract screening. Of these, 2683 were excluded at the title and abstract stage, and 44 reports were retained for full‐text assessment. All 44 reports were successfully retrieved. After full‐text review, 1 report was excluded because it did not provide eligible human cortical or directly informative post‐mortem brain transcriptomic evidence, leaving 43 studies in the final qualitative synthesis. The study‐selection workflow is summarised in Figure 1.

Studies were eligible for the core evidence base if they were peer‐reviewed primary investigations reporting human post‐mortem ASD brain transcriptomic data, with priority assigned to the cerebral cortex. Eligible transcriptomic approaches included bulk RNA sequencing, single‐cell and single‐nucleus RNA sequencing, isoform‐level analyses, alternative splicing or exon‐level studies, long‐read transcriptomic analyses and directly relevant foundational microarray studies. Integrative multi‐omic papers were included only when the ASD brain transcriptomic component could be extracted and interpreted independently. Peripheral transcriptomic studies, cerebrospinal fluid or saliva studies, organoid and induced pluripotent stem‐cell models, animal studies, imaging‐transcriptomic studies and non‐transcriptomic molecular studies were not included as core evidence. Non‐cortical human post‐mortem brain studies were considered only when they directly informed interpretation of cortical findings.

Cross‐disorder studies were included only if ASD‐specific results were reported separately. Mixed ASD cohorts were considered eligible when ASD‐related findings could be interpreted independently, whereas syndromic or highly restricted mutation‐specific studies were treated cautiously and were not allowed to define the main conclusions regarding cortical convergence. The review question was framed at the level of recurrent biological programmes rather than individual candidate genes, and final interpretation was restricted to human cortical evidence wherever possible.

For each included study, structured extraction captured cohort composition, brain region, platform, analytical design, cortical versus non‐cortical scope and major methodological limitations. Studies were then critically appraised using structured reviewer‐facing fields that distinguished direct versus partial support for the core claim, contextual‐only use, inclusion in the main table, inclusion in figure‐level synthesis, consistency with other studies, level of convergence and overall confidence. This framework was intended to prevent overstatement of the evidence, particularly in studies affected by small sample size, regional restriction, developmental dependence, cohort overlap, dataset reuse, mixed cortical and non‐cortical designs or computational reanalysis without independent biological validation.

Because the included literature was highly heterogeneous with respect to study design, tissue sampling, cellular resolution, analytical platform and cohort independence, the synthesis was narrative and evidence‐weighted rather than quantitative. Conclusions were therefore framed in terms of recurrent, but not uniform, convergence, with particular attention to where support was strongest, where it remained conditional and where apparent signals were better interpreted as contextual, indirect or potentially secondary to broader disease processes.

## 3 Transcriptomic Approaches and Interpretive Limits

The human cortical ASD transcriptomic literature is methodologically heterogeneous, and this heterogeneity defines the limits of any defensible inference about cross‐study convergence. Within the final 43‐study corpus assembled for this review, 11 studies used bulk microarray profiling, 9 used bulk RNA sequencing, 15 used single‐cell or single‐nucleus transcriptomic approaches, and 3 used single‐cell or single‐nucleus multi‐omic designs; smaller subsets focused on small‐RNA or miRNA profiling and other specialised analytical frameworks. These counts are review‐derived, but they underscore an important interpretive point: Recurrent signals in ASD cortex have emerged across analytically non‐equivalent platforms rather than within a single standardised experimental framework. Apparent recurrence should therefore not be taken to imply methodological uniformity.

Bulk‐tissue studies remain the historical and interpretive anchor of the field. Foundational microarray and RNA‐seq analyses first established the coupled pattern of neuronal or synaptic downregulation together with immune‐glial upregulation and, in several cohorts, broader disruption of regional cortical patterning (Voineagu et al. 2011; Gupta et al. 2014; Parikshak et al. 2016; Gandal et al. 2022). Their principal strength lies in the ability to capture tissue‐level programmes across large gene sets and, in the strongest studies, across more than one cortical region. At the same time, their main limitation is equally clear within the ASD literature itself: Because expression is measured across mixed cellular populations, bulk abnormalities cannot on their own distinguish altered cellular state from altered cellular composition. This is precisely why later ASD reanalyses emphasised deconvolution and composition‐sensitive interpretation rather than treating bulk signals as cell‐autonomous by default (Xu et al. 2013; Yu and He 2017). Bulk studies are thus most informative for identifying broad cortical programmes and least informative when used in isolation to assign a precise cellular origin.

Single‐cell and single‐nucleus studies materially improved cellular resolution and, in doing so, refined interpretation of the bulk signal. Across these studies, ASD‐associated dysregulation is not distributed uniformly across cortex but is enriched within defined populations, including upper‐layer excitatory neurons, subsets of interneuron‐related cells, astrocytic states and glial or microglial programmes (Velmeshev et al. 2019; Zhang, et al. 2023; Wamsley et al. 2024). This represents an important advance because it localises recurrent programmes to specific cellular contexts. Yet, the same studies also make the present limits of cell‐resolved evidence explicit. Directly generated cohorts remain modest, sampled regions remain restricted, and analytical output depends heavily on clustering, annotation and aggregation choices, particularly when nuclei rather than whole cells are profiled (Velmeshev et al. 2019; Velmeshev et al. 2020; Wamsley et al. 2024). For that reason, single‐cell and single‐nucleus studies are strongest when interpreted as localising recurrent programmes, not as demonstrating that all cortical populations or all regions are altered to the same extent.

Transcript‐level and splicing‐aware studies add a distinct inferential layer. Across the included literature, microexon dysregulation, alternative splicing, lncRNA‐associated dysregulation, allele‐specific expression, RNA editing and differential transcript usage all emerge as relevant features of ASD cortical biology (Irimia et al. 2014; Parikshak et al. 2016; Lee et al. 2019; Tran et al. 2019; Zhang et al. 2023). These studies are important because they show that convergence in ASD may occur at the level of RNA processing and transcript structure rather than solely at the level of whole‐gene abundance. At the same time, they are analytically demanding and often cohort‐sensitive: Conclusions depend more explicitly on library design, read depth, transcript annotation and validation strategy than bulk gene‐level summaries (Irimia et al. 2014; Parikshak et al. 2016; Tran et al. 2019). Their mechanistic value is therefore high, but reproducibility must still be judged case by case rather than inferred from biological specificity alone.

Small‐RNA and miRNA‐focused studies add a further post‐transcriptional layer, but here the evidence base remains comparatively thin. The available cortical studies support the plausibility of RNA‐regulatory dysregulation and link miRNA‐related changes to neuronal and immune‐associated programmes (Wu et al. 2016; Schumann et al. 2017). However, the number of such studies remains limited, and both the underlying cohorts and the analytical pipelines are more specialised than in the bulk gene‐expression literature. In the present synthesis, these studies are therefore treated as supporting evidence for transcript‐level dysregulation rather than as an equally mature line of evidence for cortical convergence.

Integrative and summary‐level studies occupy a distinct evidential position within the ASD transcriptomic literature. Cross‐platform syntheses can be highly informative when the cortical transcriptomic component remains explicit and biologically interpretable (Lombardo et al. 2017; Ramaswami et al. 2020; Velmeshev et al. 2020). However, many such studies function primarily as reanalyses or as higher‐order interpretive frameworks built on previously published datasets. This distinction is not merely procedural. Repeated appearance of a signal in the ASD transcriptomic literature does not, in itself, constitute independent replication. Within the final 43‐study corpus, only 13 studies were based on primary independent cohorts, whereas 26 relied on reused public datasets or integrative reanalysis. Recurrence across analyses should therefore not be conflated with recurrence across independent biological samples.

An additional constraint that cuts across all transcriptomic platforms is post‐mortem tissue quality. Methodological studies within the project corpus show that RNA integrity number, tissue pH and post‐mortem interval can influence transcriptomic readouts in gene‐class‐specific ways, including programmes related to neuronal function, immune response, energy metabolism and RNA processing (Stan et al. 2006; Chevyreva et al. 2008; Miyahara et al. 2023; Tian et al. 2025). This issue is particularly important in ASD because several of the recurrent disease‐associated programmes considered in this review overlap with signal classes that are themselves sensitive to tissue‐quality structure. Quality control is therefore not a detachable technical appendix, but part of the interpretive logic required to judge whether an observed cortical signal is most likely to reflect biology, confounding or some combination of both.

No single platform can therefore resolve the convergence question on its own. Bulk cortical studies provide the strongest foundation for detecting broad tissue‐level and cross‐region programmes; single‐cell and single‐nucleus studies are most informative for localising those programmes to defined cellular populations; and transcript‐aware analyses are indispensable for identifying abnormalities in RNA processing and isoform usage. The most defensible synthesis is thus one that integrates these analytical layers while preserving their different inferential scopes. Claims of cortical convergence are strongest when related programmes recur across more than one analytical layer and weakest when they depend on a single platform, a single region, a single developmental window or a repeatedly reused cohort.

## 4 Evidence Synthesis Across Core Biological Axes

Across human post‐mortem cortical transcriptomic studies, the strongest defensible inference is not that ASD is associated with a single invariant cortical expression profile. Rather, the available literature indicates that heterogeneous ASD‐related cortical states repeatedly converge, albeit non‐uniformly, on a limited set of broad biological programmes. Within the evidence‐weighting framework used here, the studies providing the strongest direct cortical anchor for this conclusion are Voineagu et al. (2011), Gupta et al. (2014), Parikshak et al. (2016), Gandal et al. (2022), Velmeshev et al. (2019), and Wamsley et al. (2024). At the same time, the evidential structure remains uneven. Only 9 of the 43 included studies were classified as providing direct support for the core convergence question, and only 13 were based on primary independent cohorts. Any defensible synthesis must therefore preserve both the recurrence of the major signals and the conditional nature of the supporting evidence. The core human post‐mortem cortical studies that most strongly inform this evidence‐weighted synthesis are summarised in Table 1, whereas the full 43‐study evidence base and reviewer‐facing appraisal fields are provided in Table S1.

**TABLE 1:** Core human post‐mortem cortical transcriptomic studies informing the evidence‐weighted synthesis of transcriptomic convergence in ASD.

st_id	Citation	Region	Platform	Cohort ASD/control	Key contribution	Main limitation
S03	Chen et al. (2020)	BA9 + BA22/41/42	Bulk	45/43	circRNA and RNA‐processing dysregulation	Reused cohort; regulatory axes largely inferred
S04	Chow et al. (2012)	DLPFC BA9/46	Bulk	15/18	Age‐dependent cortical heterogeneity	Small male‐only single‐region cohort
S05	Dias et al. (2024)	Frontal cortex	Single nucleus	13/7	Cell‐type specificity; syndromic versus idiopathic contrast	Mixed ASD cohort; dup15q‐driven signal
S06	Gandal et al. (2022)	11 cortical regions	Bulk + sn follow‐up	49/54	Multi‐cortical neuronal‐down/glial‐up convergence	Bulk‐dominant evidence; smaller cell‐type subset
S08	Ginsberg et al. (2012)	BA19	Bulk	09/09	Mitochondrial and translation‐related regional heterogeneity	Very small cortical sample; mixed cortex‐cerebellum design
S11	Gupta et al. (2014)	BA19/BA10/BA44	Bulk	32/40	Multi‐region immune‐glial upregulation plus neuronal downregulation	BA19‐weighted sampling; bulk‐based cell‐type inference
S12	Irimia et al. (2014)	BA41/42/22	Bulk/splicing	12/12	Microexon and splicing dysregulation	Selected STG subset for deep splicing analyses
S15	Lee et al. (2019)	BA9 + BA41/42/22	Bulk/ASE	56/40	Allele‐specific expression and snoRNA‐linked regulation	Specialised regulatory axis; mixed cohort structure
S18	Lombardo et al. (2017)	BA9/BA10/BA19/BA41/42/BA44	Public bulk reanalysis	NA	Hierarchical cross‐dataset cortical convergence	Entirely reused public cohorts
S20	Parikshak et al. (2016)	BA9 + STG	Bulk RNA‐seq	48/49	Large cortical RNA‐seq anchor for neuronal, glial, lncRNA and splicing changes	Prior cohort overlap and dup15q component
S21	Parras et al. (2018)	BA8/9 + BA41/42/22	Bulk + mechanistic follow‐up	54/55	CPEB4‐linked post‐transcriptional/microexon dysregulation	Human cohort reused; mechanistic depth partly from mouse work
S23	Ramaswami et al. (2020)	BA9 + BA41/42/22	Integrative multi‐omic reanalysis	48/45	Multi‐omic support for recurrent but non‐uniform convergence	No new cohort; published datasets only
S30	Tran et al. (2019)	BA9 + BA41/42/22	Bulk RNA‐seq/editing	35/34	Shared cortical RNA‐editing dysregulation	Reused discovery arm; cerebellar comparison included
S32	Velmeshev et al. (2019)	PFC + ACC	Single‐nucleus RNA‐seq	15/16	Cell‐type‐specific cortical pathology	Restricted to ACC/PFC; limited age range
S33	Velmeshev et al. (2020)	PFC/STG/BA19	Bulk RNA‐seq + ext. scRNA reference	39/46	Cross‐region, cell‐type‐informed cortical synthesis	Mixed new + reused design
S34	Voineagu et al. (2011)	BA9 + BA41/42/22 + BA44/45	Bulk microarray/RNA‐seq	16/16	Foundational convergent cortical pathology and regional attenuation	Small partly overlapping replication set; cerebellum included
S35	Wamsley et al. (2024)	BA9 ± BA4/6	snRNA‐seq + multiome	33/30	Large frontal single‐nucleus/multiomic anchor	Single‐region frontal focus; small dup15q subset
S37	Wu et al. (2016)	BA9 + BA41/42/22	Bulk miRNA + mRNA	NA	miRNA‐linked post‐transcriptional convergence	Bulk tissue only; no independent cortical replication cohort
S41	Zhang et al. (2023)	STG/BA41/42/22	Bulk + LCM neurons	27/32	Inflammatory and splicing changes in bulk and neuron‐enriched STG	Single‐region STG; QC/PMI incompleteness

Only the strongest and most interpretable studies were retained for the main table; the full reviewer‐facing evidence matrix is provided in Table S1.

## 5 Neuronal and Synaptic Programmes

The most consistently replicated axis of cortical dysregulation in ASD involves downregulation of neuronal and synaptic programmes. The primary basis for this conclusion comes from the foundational bulk cortical studies. Voineagu et al. (2011) identified a downregulated neuronal and synaptic module in frontal and temporal cortex together with an upregulated immune‐related module. Gupta et al. (2014) extended this pattern across multiple cortical regions, reporting coordinated suppression of neuronal activity‐dependent and synaptic genes alongside immune‐related activation. The same broad direction of effect was retained in the cortical RNA‐seq study of Parikshak et al. (2016), which further linked neuronal dysregulation to altered regional cortical patterning and transcript‐level abnormalities. This signal was subsequently strengthened by Gandal et al. (2022), who showed that broad neuronal and synaptic downregulation is detectable across the cerebral cortex rather than being limited to a single frontal‐temporal comparison.

Cell‐type‐resolved studies sharpen, rather than overturn, this interpretation. Velmeshev et al. (2019) showed that a substantial fraction of ASD‐associated cortical dysregulation localises to defined neuronal populations, particularly upper‐layer excitatory neurons, whereas Wamsley et al. (2024) further implicated superficial projection‐neuron populations together with glial changes. Zhang et al. (2023) provide complementary support at the neuron‐enriched level by showing that isolated superior temporal cortical neurons exhibit altered neuronal activity‐related and stress‐associated transcriptional programmes. Dias et al. (2024) are informative for a different reason: Pronounced neuronal regulatory abnormalities remain evident even in the presence of marked syndromic specificity in dup15q‐associated frontal cortex. Taken together, these studies support recurrent neuronal and synaptic dysregulation while also indicating that its precise cellular locus varies with region, cell class and cohort composition.

Additional support comes from secondary and reuse‐based analyses, although their evidential status is necessarily different. Lombardo et al. (2017) identified hierarchical cortical transcriptome disorganisation in which synaptic downregulation occupied a central position, whereas Ramaswami et al. (2020) linked depletion of neuronal and synaptic programmes to a convergent multi‐omic cortical subtype. Wu et al. (2016) add a further post‐transcriptional layer by showing that ASD‐associated miRNA dysregulation is linked to downregulated neuronal and synaptic mRNA targets. These studies are informative because they reinforce recurrence of the signal across analytical levels, but they do not carry the same inferential weight as the strongest primary cortical anchor cohorts.

The neuronal and synaptic axis is therefore most robust at the level of programme direction rather than at the level of a fixed gene list. Across studies, the specific genes, neuronal subclasses and enriched synaptic terms are not identical: Some datasets emphasise activity‐dependent transcription, others upper‐layer projection‐neuron biology, inhibitory‐neuron‐linked abnormalities or postsynaptic pathways (Voineagu et al. 2011; Gupta et al. 2014; Velmeshev et al. 2019; Wamsley et al. 2024). This variability does not weaken the central conclusion. Rather, it indicates that convergence should be described as recurrent and biologically coherent, not as molecularly identical across all cohorts.

## 6 Immune‐Glial and Neuroinflammatory Programmes

Immune‐glial and neuroinflammatory upregulation constitutes the second major axis of recurrent cortical dysregulation in ASD. The clearest support again comes from the primary anchor studies. In frontal and temporal cortex, Voineagu et al. (2011) identified an astrocyte‐ and microglia‐associated module that was upregulated in parallel with neuronal downregulation. Gupta et al. (2014) extended this pattern across multiple cortical regions, reporting coordinated elevation of innate immune, microglial and astrocytic programmes. This broad immune‐related direction was retained in the larger RNA‐seq framework of Parikshak et al. (2016) and was further strengthened by Gandal et al. (2022), who showed that glial and neural‐immune upregulation is detectable across multiple cortical areas rather than being confined to a single regional comparison.

Cell‐resolved studies sharpen this interpretation by showing that the immune signal is not adequately explained by bulk‐tissue enrichment alone. Velmeshev et al. (2019) identified altered microglial and astrocytic states in parallel with neuronal vulnerability, whereas Wamsley et al. (2024) likewise described reactive astrocytic, oligodendroglial and microglial programmes in ASD cortex. Velmeshev et al. (2020) provide additional cross‐region support by linking inflammatory and cell‐type‐informed signals across ASD neocortex, although the evidential status of that study is necessarily mixed because part of the analysis depends on reprocessed rather than wholly independent cortical data. Zhang et al. (2023) add a further nuance by showing that inflammatory or stress‐related transcriptional signatures are detectable within isolated ASD neurons, raising the possibility that neuronal stress and glial activation are transcriptionally coupled rather than fully separable processes.

What remains unresolved is not the presence of this axis, but its precise biological interpretation. Across studies, the signal is variably expressed as microglial and astrocytic activation, interferon‐related transcription, complement and defence‐response pathways or broader inflammatory enrichment (Voineagu et al. 2011; Gupta et al. 2014; Gandal et al. 2022). In syndromic or subgroup‐specific contexts, this component may be particularly prominent, as illustrated by dup15q‐associated cortex in Dias et al. (2024). This ambiguity is biologically important because microglia are not limited to inflammatory reactivity alone; under physiological developmental conditions, they also contribute to circuit refinement, including activity‐ and complement‐dependent synaptic pruning (Paolicelli et al. 2011; Schafer et al. 2012). Microglial transcriptional changes in ASD cortex should therefore not be assumed to denote a single stereotyped pathological state. Depending on developmental context and the accompanying degree of neuronal stress, they may reflect reactive immune activation, altered homeostatic glial functions or disruption of developmental microglial roles that normally contribute to synaptic maturation. The available literature therefore supports robust immune‐glial convergence at the level of broad programme architecture, but not a single uniform inflammatory state shared across all cortical regions, cell types or ASD subgroups.

The strongest defensible inference is therefore limited but clear. Human cortical transcriptomic studies recurrently identify upregulated neuroimmune and glial programmes that accompany neuronal and synaptic downregulation across multiple ASD cohorts. However, current transcriptomic evidence does not determine whether these glial changes represent primary drivers, reactive responses or bidirectional amplifiers of cortical pathology. The recurrent signal is well supported; its causal ordering is not.

## 7 RNA Processing, Splicing and Transcript‐Level Regulation

A third major axis of cortical dysregulation in ASD involves altered RNA processing and transcript‐level regulation. Support for this branch is substantial and cannot be regarded as a marginal observation confined to a small number of specialised studies. Voineagu et al. (2011) already implicated abnormal alternative splicing linked to RBFOX‐ or A2BP1‐associated regulation. This framework was extended by Parikshak et al. (2016), who identified widespread splicing alterations together with lncRNA dysregulation and attenuation of frontal‐temporal regional identity. Particularly strong direct support was provided by Irimia et al. (2014), who demonstrated recurrent misregulation of a highly conserved neuronal microexon programme associated with reduced nSR100 or SRRM4.

Subsequent studies broadened this transcript‐level branch well beyond exon inclusion alone. Parras et al. (2018) linked ASD‐relevant post‐transcriptional dysregulation to CPEB4 mis‐splicing and altered deadenylation, although the human cortical component of that study was coupled to mechanistic follow‐up outside the primary human tissue. Lee et al. (2019) identified allele‐specific expression abnormalities with a more specialised transcript‐regulatory profile. Tran et al. (2019) reported widespread cortical RNA hypoediting across ASD brains, whereas Chen et al. (2020) described robust circRNA dysregulation and circRNA–miRNA–mRNA network abnormalities, although much of the regulatory architecture in that study remains computationally inferred. Zhang et al. (2023) further strengthened this branch by identifying abnormalities related to the spliceosome, snoRNA biology and mRNA metabolism in superior temporal cortex and isolated neurons.

What unifies these studies is not a single universal transcriptomic lesion, but repeated evidence that ASD‐associated cortical dysregulation extends beyond total gene abundance into exon choice, isoform usage, RNA editing, circRNA biology and other post‐transcriptional mechanisms (Irimia et al. 2014; Parikshak et al. 2016; Tran et al. 2019; Chen et al. 2020; Zhang et al. 2023). This is one of the clearest reasons that gene‐level summaries alone are insufficient for interpreting ASD cortical biology. At the same time, this branch remains more analytically conditional than the broad neuronal/synaptic and immune‐glial axes. Many of the key signals depend on region‐restricted cohorts, specialised computational pipelines, selected sample subsets or reused datasets. The strongest defensible inference is therefore that altered transcript‐level regulation represents a major recurrent dimension of ASD cortical pathology, whereas the reproducibility of any individual exon‐, isoform‐ or editing‐level event remains more contingent.

## 8 Mitochondrial and Metabolic Signals as a Linked but Less Consolidated Layer

Mitochondrial and broader metabolic signals recur across the literature, but this branch is less consolidated than the neuronal/synaptic, immune‐glial and RNA‐processing axes. Ginsberg et al. (2012) identified abnormalities related to oxidative phosphorylation and protein translation in a small cohort spanning occipital cortex and cerebellum. Schwede et al. (2018) reported coordinated downregulation of synaptic and mitochondrial genes in reanalysed cortex. Ramaswami et al. (2020) linked mitochondrial neuronal programmes to a convergent molecular subtype, and Zhang et al. (2023) identified mitochondrial and ribosomal downregulation in superior temporal cortex together with broader abnormalities involving neuronal stress and RNA processing.

These findings are biologically plausible, but their evidential footing is weaker than that of the three principal axes. Relative to the strongest anchor studies, mitochondrial signals more often emerge from small cohorts, mixed cortical and non‐cortical designs, region‐restricted sampling or reuse‐heavy reanalysis frameworks (Ginsberg et al. 2012; Schwede et al. 2018; Ramaswami et al. 2020). They are also less consistently foregrounded in the highest‐confidence direct cortical studies, which centre more clearly on neuronal/synaptic, immune‐glial, regional‐patterning and transcript‐regulatory abnormalities (Voineagu et al. 2011; Parikshak et al. 2016; Gandal et al. 2022; Wamsley et al. 2024). On that basis, mitochondrial‐related dysregulation is best interpreted as a linked and repeatedly observed associated layer rather than as an equally well‐established core programme of cortical convergence.

## 9 Integrated Interpretation

Taken together, the available literature supports a tiered model of cortical transcriptomic convergence in ASD rather than a single unitary molecular signature. The most robust recurrent pattern consists of coupled neuronal and synaptic downregulation together with immune‐glial upregulation, a conclusion that is strongest when the primary cortical anchor studies are considered jointly and then refined by cell‐type‐resolved analyses. A second layer, also well supported but more analytically conditional, involves abnormalities in RNA processing, alternative splicing, isoform regulation, RNA editing and related post‐transcriptional mechanisms. By contrast, mitochondrial and broader metabolic signals remain biologically relevant and repeatedly observed, but are less consolidated within the strongest human cortical evidence base. Across all four axes, however, the apparent consistency of the literature is moderated by region, developmental stage, cohort structure, cell‐type resolution and dataset independence.

**FIGURE 2:**
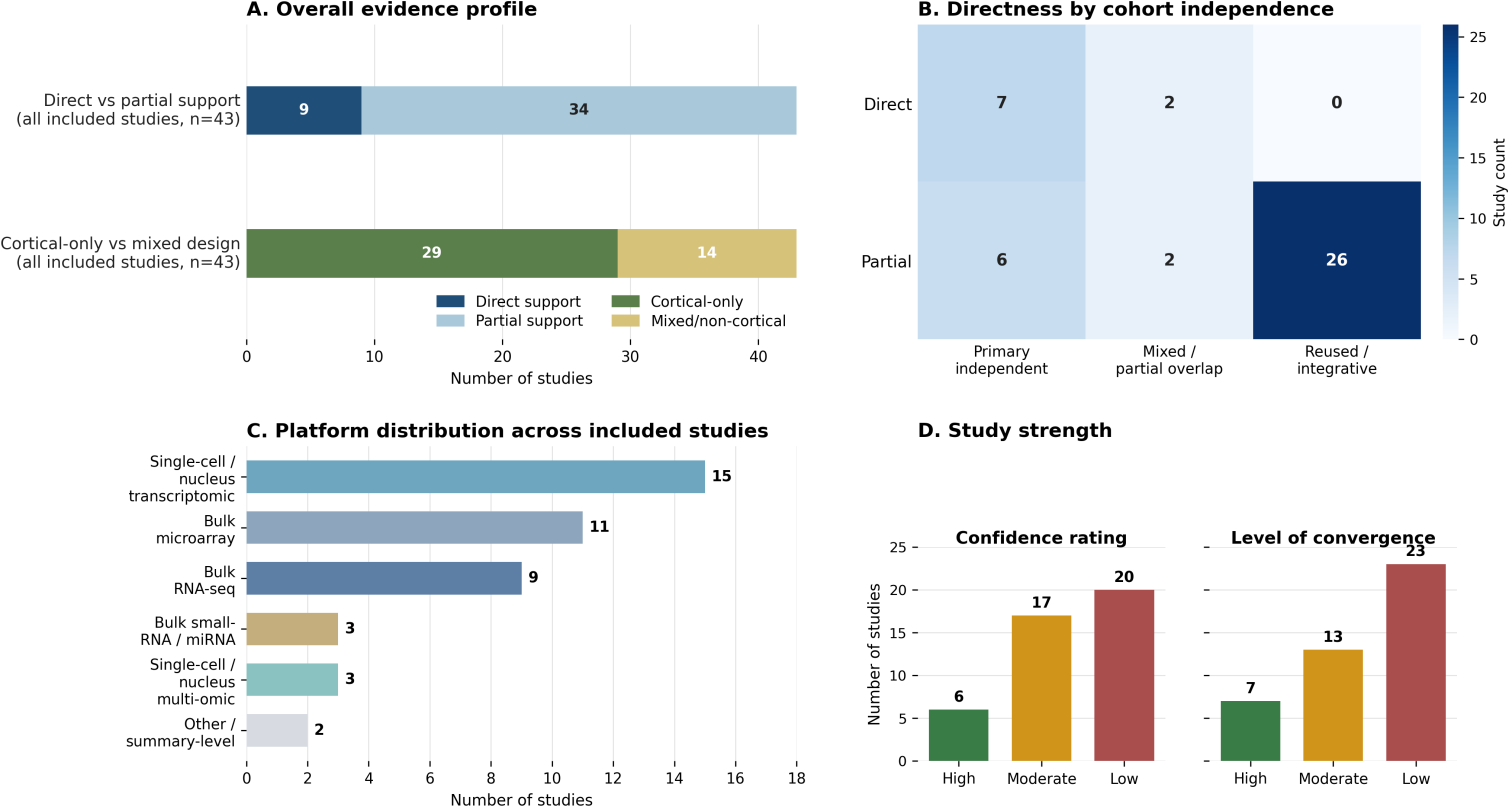
Semi‐quantitative evidence map of the included human post‐mortem ASD cortical transcriptomic literature. Panel A summarises the overall evidence profile across all 43 included studies, showing direct versus partial support for the core review question and cortical‐only versus mixed/non‐cortical study design. Panel B shows the relationship between directness of support and cohort independence. Panel C summarises platform distribution across the included literature. Panel D shows overall confidence rating and level of convergence across included studies. Counts are derived from the reviewer‐facing 43‐study evidence matrix used for this review.

The most conservative and defensible synthesis is therefore not that ASD cortex is characterised by a single transcriptomic lesion. Rather, the evidence supports recurrent cortical convergence at the level of a limited set of broad biological axes whose expression is probabilistic, context dependent and not uniformly reproduced across independent cohorts. This is sufficient to justify a critical convergence framework, but not to erase the heterogeneity that remains clearly visible across the human cortical literature. A semi‐quantitative overview of the structure of the included evidence base is shown in Figure 2.

## 10 Causality Versus Consequence

Any causal interpretation of ASD cortical transcriptomics must begin with the structure of the evidence itself. The human cortical datasets considered here are post‐mortem and overwhelmingly postnatal, whereas the strongest developmental and genetic evidence for ASD implicates earlier vulnerability in fetal cortical systems involved in neuronal differentiation, projection‐neuron development, synaptic maturation and gene regulation (Willsey et al. 2013; Garcia‐Forn et al. 2020; Courchesne et al. 2020; Satterstrom et al. 2020). For that reason alone, current cortical transcriptomic studies are better suited to defining recurrent downstream pathological programmes than to identifying the earliest point of mechanistic divergence. The present literature can therefore support graded causal plausibility, but not direct temporal proof.

Within this framework, neuronal‐developmental, synaptic and gene‐regulatory abnormalities lie closest to primary ASD liability. This inference does not depend on any single study, but on convergence between developmental genetics and the strongest cortical anchor studies. Human genetic and developmental work places substantial ASD liability in fetal cortical systems enriched for neuronal communication, cortical projection‐neuron development and regulatory programmes (Willsey et al. 2013; Sullivan et al. 2019; Garcia‐Forn et al. 2020; Courchesne et al. 2020; Satterstrom et al. 2020). The cortical transcriptomic literature is broadly aligned with that architecture: Voineagu et al. (2011), Gupta et al. (2014), Parikshak et al. (2016), Velmeshev et al. (2019), Gandal et al. (2022) and Wamsley et al. (2024) repeatedly identify neuronal and synaptic downregulation, altered cortical patterning and cell‐type‐specific neuronal vulnerability, rather than merely non‐specific terminal stress signatures. These observations do not establish that the measured cortical expression changes are themselves causal. They do, however, place neuronal and gene‐regulatory dysregulation closer to primary liability than many of the other recurrent signals observed in mature post‐mortem tissue.

Altered RNA processing likely occupies a relatively proximal position within this same framework, albeit with greater analytical caution. Across the cortical evidence base, microexon dysregulation, alternative splicing, transcript usage, circRNA biology and RNA editing recur across multiple studies rather than appearing as isolated curiosities (Voineagu et al. 2011; Irimia et al. 2014; Parikshak et al. 2016; Parras et al. 2018; Tran et al. 2019; Chen et al. 2020; Zhang et al. 2023). This is important because transcript‐regulatory abnormalities can plausibly intersect with neuronal maturation and cortical circuit assembly more directly than broad metabolic signatures. At the same time, post‐mortem transcriptomic data alone cannot determine whether any given RNA‐processing abnormality is primary, modifying or downstream. The most defensible formulation is therefore that RNA‐processing abnormalities are mechanistically informative and potentially proximal, but remain correlational within the present human cortical evidence base.

Immune‐glial programmes occupy a different inferential position: Their recurrence in ASD cortex is well supported, but their temporal placement remains unresolved. Across the major bulk cortical anchor studies, immune and glial upregulation emerges as a recurrent feature, and this signal is further sharpened by cell‐type‐resolved work showing altered astrocytic, microglial and oligodendroglial states (Voineagu et al. 2011; Gupta et al. 2014; Parikshak et al. 2016; Velmeshev et al. 2019; Gandal et al. 2022; Wamsley et al. 2024). This body of evidence makes the existence of immune‐glial dysregulation difficult to dismiss. At the same time, it does not determine whether glial activation is primary in some contexts, secondary to neuronal dysfunction in others or part of a bidirectional pathological loop. That uncertainty is increased by the possibility that later cortical neuroimmune states are shaped not only by intracortical processes but also by systemic and developmental influences that are only indirectly visible in post‐mortem tissue. Gene–environment and neuroimmune studies support the plausibility that prenatal inflammatory exposures, broader environmental factors and microbiota‐dependent gut–brain signalling that influences microglial maturation and function may condition later central immune states (Erny et al. 2015; Abdel‐Haq et al. 2019; Cheroni et al. 2020; Han et al. 2021). In ASD, however, these pathways are better treated as plausible modifiers rather than established explanations of the cortical transcriptomic signal, because current human post‐mortem datasets do not resolve their timing, magnitude, specificity or direction of effect. Thus, the signal itself is robust, whereas its temporal and causal ordering remains unresolved.

Greater caution is required for mitochondrial and broader metabolic signals. Although these abnormalities recur across the literature, they are less consolidated within the highest‐confidence cortical anchor studies and more often emerge from small cohorts, mixed cortical and non‐cortical designs or reuse‐heavy analytical frameworks (Ginsberg et al. 2012; Schwede et al. 2018; Ramaswami et al. 2020; Zhang et al. 2023). For that reason, mitochondrial abnormalities cannot be framed as established primary drivers on the basis of human cortical transcriptomics alone. Within the present evidence base, they are better interpreted as biologically relevant but more conditional layers, potentially reflecting downstream adaptation, altered energetic demand or other secondary features of already established cortical dysfunction.

The most defensible causal framework is therefore hierarchical rather than binary. Current human cortical transcriptomics most strongly supports a relatively proximal layer involving neuronal‐developmental, synaptic and transcript‐regulatory dysregulation, consistent with ASD genetic architecture and fetal cortical vulnerability. A second layer of immune‐glial dysregulation is robustly present but cannot yet be confidently assigned as primary, secondary or bidirectionally amplifying. A third layer of mitochondrial and metabolic change is repeatedly observed, but currently rests on a weaker and less independent evidential base. Human post‐mortem cortex does not permit definitive separation of cause from consequence. It does, however, justify a more disciplined formulation in which some recurrent programmes are more plausibly upstream‐facing than others.

## 11 Transcriptomic Convergence and ASD Genetic Architecture

The relation between cortical transcriptomic convergence and ASD genetic architecture is most appropriately interpreted at the level of biological programmes rather than as a one‐to‐one correspondence between risk genes and differentially expressed genes. ASD liability is genetically heterogeneous, encompassing both rare high‐impact variation and diffuse common polygenic risk (Grove et al. 2019; Satterstrom et al. 2020). Under such an architecture, a single universal risk‐gene‐expression signature would not be expected in human post‐mortem cortex. A more plausible expectation is that heterogeneous upstream liability converges on a restricted set of developmental and cellular pathways. It is at this level that the current cortical transcriptomic literature is most informative.

The clearest point of contact between genetics and transcriptomics lies in neuronal‐developmental, synaptic and gene‐regulatory programmes. Independent developmental‐genetic studies place substantial ASD liability in fetal cortical systems involved in neuronal differentiation, projection‐neuron‐related development, synaptic communication and regulatory programmes (Willsey et al. 2013; Sullivan et al. 2019; Garcia‐Forn et al. 2020; Courchesne et al. 2020; Satterstrom et al. 2020). The highest‐confidence cortical transcriptomic anchor studies are broadly concordant with this architecture. Across different analytical frameworks, Voineagu et al. (2011), Parikshak et al. (2016), Velmeshev et al. (2019), Gandal et al. (2022) and Wamsley et al. (2024) repeatedly implicate neuronal and synaptic dysregulation, altered cortical patterning, cell‐type‐specific neuronal vulnerability and transcript‐regulatory disruption. This does not indicate that post‐mortem cortical expression directly recapitulates the earliest developmental action of ASD risk variation. It does indicate that the dominant cortical transcriptomic signals are aligned with, rather than disconnected from, the principal neurodevelopmental axes implicated by human genetics.

Several studies make this connection more explicit by linking dysregulated cortical programmes to ASD‐risk‐enriched regulatory space. In the foundational cortical literature, module‐level disorganisation was already interpreted in relation to autism candidate genes (Voineagu et al. 2011). Parikshak et al. (2016) extended that framework by linking altered cortical transcription, lncRNA regulation and splicing abnormalities to ASD‐relevant biology. Parras et al. (2018) further suggested that CPEB4‐dependent post‐transcriptional dysregulation may affect a substantial fraction of high‐confidence ASD risk gene transcripts. Chen et al. (2020) connected cortical circRNA‐associated regulatory axes to ASD risk genes, and Wamsley et al. (2024) provided particularly strong cell‐type‐resolved support by reporting enrichment of ASD‐associated regulons and their drivers in both rare and common genetic risk. Taken together, these studies support a meaningful, but not isomorphic, intersection between ASD genetic architecture and cortical transcriptomic convergence, particularly within neuronal and transcript‐regulatory domains.

This alignment is not uniform across all recurrent transcriptomic signals. Genetic anchoring is strongest for neuronal, synaptic, developmental and transcript‐regulatory branches. It is less direct for the immune‐glial axis and weaker still for mitochondrial and broader metabolic signals. This asymmetry is important. Recurrent immune and glial dysregulation in cortex is well supported transcriptomically, but that does not by itself establish glial programmes as the principal site of inherited ASD liability. Likewise, mitochondrial and metabolic abnormalities may be biologically important without being equally proximal to the gene‐regulatory and neuronal‐developmental pathways most strongly implicated by human genetics. Genetic architecture therefore does not confer equal mechanistic status on every recurrent cortical transcriptomic abnormality.

The contrast between rare and common variation further argues against simplified claims of uniform convergence. Rare high‐impact variants can generate strong, and at times syndromic, molecular signatures, whereas common risk is more diffuse and likely to perturb networks rather than single dominant targets (Grove et al. 2019; Satterstrom et al. 2020). The cortical transcriptomic literature is consistent with this model. Syndromic contexts such as dup15q‐associated cortex can exhibit marked syndrome‐specific signatures while still retaining partial overlap with broader ASD‐related neuronal and regulatory programmes (Parikshak et al. 2016; Dias et al. 2024). This pattern is important because it indicates that transcriptomic convergence in ASD is compatible with etiological diversity: Distinct forms of genetic liability may intersect at shared cortical programmes without producing identical whole‐transcriptome states.

Important limits remain on what can be inferred from the current human cortical datasets. Most transcriptomic studies do not integrate donor‐level genomic data in a manner that permits direct genotype‐to‐expression attribution. Many of the strongest cortical datasets are derived from childhood‐to‐adult post‐mortem tissue rather than the prenatal windows highlighted by developmental genetics. Repeated reuse of the same public cohorts also means that enrichment signals are not always evaluated in fully independent transcriptomic samples. Cortical transcriptomic convergence should therefore not be presented as a direct readout of ASD genetic architecture. A more defensible formulation is narrower: Human genetics and human cortical transcriptomics converge most clearly on neuronal‐developmental, synaptic and transcript‐regulatory programmes, whereas immune‐glial and, especially, mitochondrial‐metabolic signals remain less directly anchored to the principal inherited‐risk framework.

## 12 Limitations of the Evidence Base

The available human cortical transcriptomic literature in ASD is informative, but it is not methodologically uniform, and the structure of that evidence should define the strength of every conclusion drawn from it. The first major constraint is limited cohort independence. Of the 43 included studies, only 9 were judged to provide direct support for the core convergence question, and only 13 were based on primary independent cohorts, whereas 26 relied on reused public datasets or integrative reanalysis and 4 involved mixed or partially overlapping cohort structures. This is not a minor procedural issue. Some of the most influential themes in the field have been reinforced repeatedly within a relatively narrow set of source cohorts rather than across many fully independent cortical samples (Xu et al. 2013; Lombardo et al. 2017; Yu and He 2017; Ramaswami et al. 2020; Velmeshev et al. 2020). Repeated appearance of a signal should therefore not be taken automatically to represent independent biological replication.

A second major limitation is uneven biological sampling. Much of the literature is concentrated in selected association‐cortex regions, particularly frontal, prefrontal, anterior cingulate and superior temporal cortex, whereas large portions of cortex remain 13. post‐mortem tissue rather than the prenatal and very early postnatal intervals most strongly implicated by ASD genetics and cortical developmental models (Willsey et al. 2013; Garcia‐Forn et al. 2020; Courchesne et al. 2020). In addition, several studies are small, sex‐imbalanced, region‐restricted or combine idiopathic and syndromic contexts, as illustrated by both older cortical cohorts and newer subgroup‐specific work such as dup15q‐associated cortex (Ginsberg et al. 2012; Velmeshev et al. 2019; Dias et al. 2024). For that reason, absence of a given signal in one cohort should not be read straightforwardly as evidence against convergence, just as a strong signal in one subgroup should not be generalised uncritically to all ASD cortex.

A third limitation lies in the analytical non‐equivalence of the available platforms. Bulk microarray, bulk RNA‐seq, single‐cell and single‐nucleus sequencing, small‐RNA profiling and transcript‐level or isoform‐focused approaches do not capture identical biological features and are not directly interchangeable. Bulk studies are well suited to detecting broad tissue‐level programmes, but they cannot cleanly distinguish within‐cell dysregulation from shifts in cellular composition. This is precisely why later reanalyses emphasised composition‐sensitive interpretation and deconvolution rather than a naive cell‐autonomous reading of bulk results (Voineagu et al. 2011; Xu et al. 2013; Yu and He 2017). Cell‐resolved studies localise dysregulation more effectively, but they are typically based on smaller donor numbers, more restricted regional sampling and analytically complex clustering and annotation pipelines (Velmeshev et al. 2019; Wamsley et al. 2024). Transcript‐level studies add substantial mechanistic value, particularly for RNA processing but depend more heavily on sequencing depth, library design and specialised computational analysis (Irimia et al. 2014; Parikshak et al. 2016; Tran et al. 2019; Zhang et al. 2023). Apparent inconsistency across studies may therefore reflect true biological heterogeneity, platform‐specific visibility or some combination of both.

Post‐mortem tissue quality imposes a fourth major constraint. RNA integrity, tissue pH, post‐mortem interval, agonal state and related terminal variables can influence broad gene‐expression programmes, including neuronal, immune‐related, metabolic and RNA‐processing signals (Stan et al. 2006; Chevyreva et al. 2008; Miyahara et al. 2023; Tian et al. 2025). These are not peripheral technical nuisances, because they overlap with precisely the biological programmes most often emphasised in the ASD cortical literature. Although the stronger ASD studies address such factors carefully, the quality structure of post‐mortem tissue cannot be treated as fully separable from biological interpretation, particularly in small cohorts, older microarray‐era datasets or low‐RIN material (Voineagu et al. 2011; Ginsberg et al. 2012; Gupta et al. 2014).

A further limitation is that cortical transcriptomics remains fundamentally correlational. Even where recurrent neuronal, immune‐glial or transcript‐regulatory signals are well supported, post‐mortem cortex does not establish temporal ordering. It cannot, on its own, determine whether a programme is primary, compensatory, secondary or bidirectionally amplified over time. This point is especially important because the strongest developmental‐genetic evidence implicates earlier fetal cortical vulnerability than the tissue windows represented in most transcriptomic cohorts (Willsey et al. 2013; Garcia‐Forn et al. 2020; Courchesne et al. 2020; Satterstrom et al. 2020). Mature post‐mortem cortex is therefore more likely to capture accumulated downstream molecular states than the earliest point of divergence.

The design of the present review also defines the scope of inference. This article was structured as a cortex‐centred evidence synthesis with systematic database searching and transparent study‐level weighting, rather than as a statistical meta‐analysis intended to generate pooled effect sizes or formal quantitative estimates of reproducibility. Screening and appraisal were performed by a single reviewer, but explicit eligibility criteria and reviewer‐facing evidence fields were used to keep study selection and interpretive weighting transparent. The deliberate emphasis on human cortical tissue also means that peripheral, organoid and animal‐model studies are treated selectively, mainly where they clarify interpretation, whereas the central synthesis remains anchored to post‐mortem cortical evidence.

Taken together, these limitations justify a deliberately conservative interpretation. The current literature is sufficient to support recurrent, but not uniform, convergence on a limited number of cortical biological programmes. It is not sufficient to justify claims of a single invariant transcriptomic signature, fully resolved causal ordering or complete generalisability across cortical regions, developmental windows, cell types and ASD subgroups. Framed in this way, the limitations do not diminish the value of the evidence base. Rather, they define the level of confidence that it can presently sustain.

## Conclusion

The currently available human post‐mortem cortical transcriptomic literature in ASD does not support a single invariant molecular signature of cortex. Rather, the most interpretable studies indicate recurrent, though non‐uniform, convergence on a restricted set of biological programmes. At the centre of this pattern lies coupled neuronal and synaptic dysregulation together with immune‐glial activation. Superimposed on this is a substantial transcript‐regulatory layer encompassing altered splicing, isoform‐level regulation, RNA editing and related post‐transcriptional processes.

At the same time, the apparent strength of this convergence cannot be separated from the structure of the evidence base on which it rests. Only a minority of studies provide direct support for the core cortical convergence question; much of the literature depends on repeated analysis of a limited number of source cohorts; regional and developmental sampling remain uneven; and many reported signals remain contingent on platform, cellular resolution and post‐mortem quality structure. The most defensible interpretation is therefore not that ASD cortex is defined by a unified transcriptomic lesion but that heterogeneous ASD‐related cortical states repeatedly intersect at a limited number of broad biological axes whose expression is probabilistic, context dependent and not fully independent across studies.

The conclusion of this review is accordingly intended to be conservative. Human cortical transcriptomics is already sufficiently mature to support a critical convergence framework centred on neuronal‐developmental, synaptic, immune‐glial and transcript‐regulatory biology. It is not yet sufficiently mature to justify universal claims, definitive causal ordering or the treatment of all recurrent signals as equally anchored to ASD genetic architecture. The current literature does not provide a final molecular definition of ASD cortex. It does, however, provide a disciplined map of the biological programmes that recur most consistently across the currently available human post‐mortem cortical studies.

## Funding

The author has nothing to report.

## Ethics Statement

Ethical approval was not required for this study because it is a review of previously published literature and did not involve direct research on human participants or animals.

## Consent

Patient consent was not required for this study because it did not involve identifiable human participants or patient data.

## Conflicts of Interest

The author declares no conflicts of interest.

## Supporting information


**Table S1:** Supporting Information.

## Data Availability

The data supporting the findings of this study are available within the article and its Supporting Information.
